# Supplementary material for: Identification of photoperiod-induced specific miRNAs in the adrenal glands of Sunite sheep (Ovis aries)
Source: Front Vet Sci. 2022 Jul 22;9:888207. doi: 10.3389/fvets.2022.888207 (PMC9354845; doi:10.3389/fvets.2022.888207)
Supplement: Supplementary file 1 [file Data_Sheet_1.ZIP › Supplementary file/miRDeep2 file.zip!/miRDeep2 file/Novel_Structure/SPLP42A1/Novel_521.pdf]

Provisional ID	:	NC_019460.2_6165
Score total	:	0.8
Score for star read(s)	:	-1.3
Score for read counts	:	-1.3
Score for mfe	:	2
Score for randfold	:	1.6
Score for cons. seed	:	
Total read count	:	9
Mature read count	:	9
Loop read count	:	0
Star read count	:	0

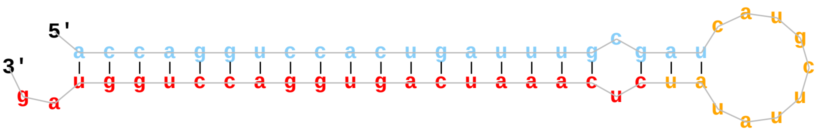

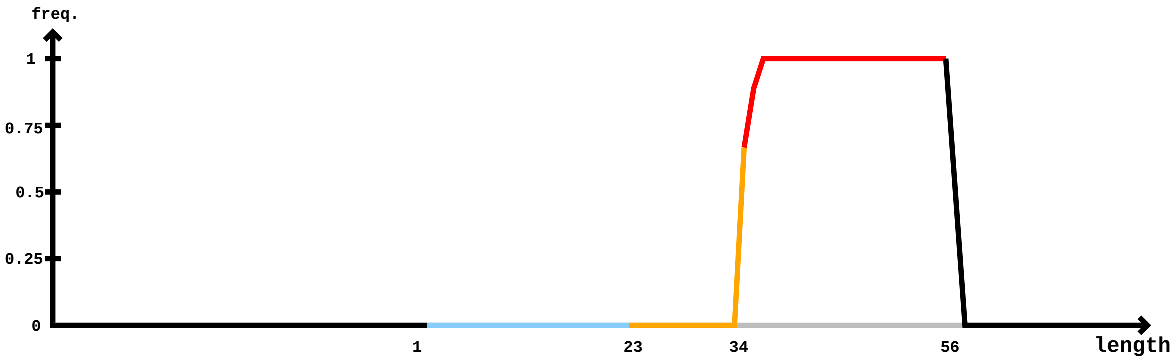


```
                                                 Star                                      Mature
5'- uaacaauaauaauagaccaugagagaucuauaaaucaaccagguccacugauuugcgaucaugcuuauaucucaaaucaguggaccugguagauuuacuguauuuuuguaug   -3'    exp
    .................(((((((((((..((((((.((((((((((((((((((.(((.........))).)))))))))))))))))))).))))))..).)))))).))))    reads     mm         sample
    ......................................................................cucaaaucaguggaccugguag....................    5         0          seq
    ......................................................................cucaaaucaguggaccugguaga...................    1         0          seq
    .......................................................................ucaaaucaguggaccugguaga...................    2         0          seq
    ........................................................................caaaucaguggaccugguaga...................    1         0          seq
```
